# Projected life-year gains with semaglutide in individuals with cardiovascular disease without type 2 diabetes in the UK

**DOI:** 10.1530/EC-25-0903

**Published:** 2026-03-23

**Authors:** Thomas Van Sloten, Steven H J Hageman, Jan Westerink, Silvia Capucci, João Diogo da Rocha Fernandes, Sara Holloway, Andrew Pijper, Natalie Tikhonovsky, Scott Emerson, A Michael Lincoff, Jorge Plutzky, Ildiko Lingvay

**Affiliations:** ^1^ Department of Vascular Medicine, Diabetology and Endocrinology, University Medical Center Utrecht, Utrecht, Netherlands; ^2^ Department of Internal Medicine, Isala Clinics, Zwolle, Netherlands; ^3^ Novo Nordisk A/S, Søborg, Denmark; ^4^ Lane Clark & Peacock LLP, London, UK; ^5^ Washington State Hospital Association, Seattle, Washington, USA; ^6^ Department of Biostatistics, University of Washington, Seattle, Washington, USA; ^7^ Department of Cardiovascular Medicine, Cleveland Clinic, Cleveland Clinic Lerner College of Medicine, Case Western Reserve University, Cleveland, Ohio, USA; ^8^ Division of Cardiovascular Medicine, Brigham and Women’s Hospital, Harvard Medical School, Boston, Massachusetts, USA; ^9^ Division of Endocrinology, Department of Internal Medicine, University of Texas Southwestern Medical Center, Dallas, Texas, USA; ^10^ Peter O’Donnell Jr. School of Public Health, University of Texas Southwestern Medical Center, Dallas, Texas, USA

**Keywords:** SELECT, semaglutide, glucagon-like peptide-1 receptor agonist, cardiovascular disease, overweight/obesity

## Abstract

In the SELECT cardiovascular (CV) outcomes trial, semaglutide 2.4 mg significantly reduced all-cause mortality or non-fatal CV events versus placebo in patients with CV disease (CVD) and overweight/obesity, but without type 2 diabetes (T2D). By applying results from SELECT to a UK real-world population, this present cohort study evaluated the impact of semaglutide on life expectancy and CV event-free life-years. Included were individuals from the Discover electronic health record database who met SELECT eligibility criteria (≥45 years old; body mass index: ≥27 kg/m^2^; established CVD; no T2D). The Discover database comprises linked primary and secondary care data covering people residing in North West London, UK. Cohort entry was the first date between 2004 and 2019 when all inclusion criteria were met. Effect estimates from SELECT were applied using actuarial life tables, estimating the effect of semaglutide on life expectancy overall and by age group. Life expectancy gains and CV event-free life expectancy were estimated using the Arriaga and Sullivan methods, respectively. In total, 19,117 individuals aged ≥45 years were included in the main analysis (63% men; mean age: 65.4 years). The estimated gain in life expectancy with semaglutide treatment was 1.9 years. A CV event-free life-year gain of 2.0 years was predicted with semaglutide. Life-year gains were observed across all age groups but were higher for younger age groups. Based on SELECT data, semaglutide is predicted to increase life expectancy and CV event-free life-years in a real-world population with overweight/obesity and CVD, particularly in younger people.

## Introduction

Life expectancy has declined worldwide from 2020 to 2025 (1). Global life expectancy and healthy life expectancy at birth in 2021 was 71.4 and 61.9 years, respectively, which is a decline to levels last seen in 2012 (1). In the UK, life expectancy for men and women aged 65 years between 2021 and 2023 was 18.5 and 21.0 years, respectively; this is a decline by 9 weeks for men and 5 weeks for women compared with 2017–2019 (2). Similar trends have been observed in the USA (3). Since 2020, changes in life expectancy reductions have been partially affected by the COVID-19 pandemic, which has been associated with cases of multiple organ failure (4). The other main factors driving decreases in life expectancy are chronic diseases and multimorbidity, with ischaemic heart disease the leading cause of death globally in 2021 (5) and cardiovascular disease (CVD) the second largest contributor to years of life lost in the UK in 2019 (6). The prevalence of obesity has increased rapidly, and individuals with obesity have a significantly higher risk of all-cause mortality compared with those with healthy weight (7, 8, 9).

Compared with people with healthy weight, people living with overweight or obesity are at an increased risk of developing CVD and other chronic comorbidities (10, 11). In 2022, the World Health Organization estimated that 43 and 16% of adults were classified as having overweight (body mass index (BMI): 25–<30 kg/m^2^) and obesity (BMI: ≥30 kg/m^2^), respectively (12). Compared with groups with healthy weight, the risk of all-cause mortality in these groups increased by 31% per 5 kg/m^2^ difference in BMI for all individuals and by 42% for individuals with coronary heart disease or stroke (10). Similarly, in a US population, statistically significant risks of all-cause mortality were observed in individuals with a BMI ≥30 kg/m^2^ compared with those with healthy weight (13). These global trends of rising BMI suggest that obesity may be contributing to changes in life expectancy. This highlights a need for appropriate weight management and cardiovascular (CV) risk reduction in people with overweight or obesity.

The SELECT CV outcomes trial (NCT03574597) assessed the efficacy and safety of the glucagon-like peptide-1 receptor agonist (GLP-1RA) semaglutide at a once weekly dose of 2.4 mg versus placebo, both as an adjunct to standard of care, in patients aged ≥45 years with established CVD and overweight or obesity, but without type 2 diabetes (T2D) (14). In this trial, semaglutide was associated with reductions in the incidence of major adverse CV events (CV death, non-fatal myocardial infarction, or non-fatal stroke; hazard ratio (HR): 0.80; 95% confidence intervals (CI): 0.72–0.90) and all-cause mortality (HR: 0.81; 95% CI: 0.71–0.93) versus placebo. However, it remains unclear to what degree treatment with semaglutide can increase life expectancy and years lived without experiencing a recurrent CV event in a real-world setting. Here, we estimate the effect of semaglutide on life expectancy and recurrent CV event-free life-years by applying results from the SELECT trial to a real-world population meeting the SELECT eligibility criteria.

## Methods

### Data source

This open cohort study used data from the Discover electronic health record database, which comprises linked primary and secondary electronic health records covering 2.7 million people residing in North West London, UK (15). The Discover dataset has a similar age and sex distribution to the UK general population but is ethnically more diverse (15). Primary care data were available from 1 January 2004; secondary care data were available from 1 January 2015.

### Study population

Individuals from Discover who met the SELECT trial criteria (≥45 years old with a BMI ≥27 kg/m^2^, established CVD, and no history of T2D) were included. BMI was either derived from direct measurements or calculated from available weight and height measurements. As in the SELECT trial, established CVD, identified by International Classification of Diseases, Tenth Revision (ICD-10), diagnosis codes, was defined as a history of myocardial infarction (MI), stroke, or symptomatic peripheral artery disease with peripheral arterial revascularisation procedure, claudication with ankle–brachial index <0.85, or amputation due to atherosclerotic disease.

Excluded were individuals with glycated haemoglobin (HbA_1c_) ≥48 mmol/mol or a history of diabetes or those receiving treatment with any glucose-lowering agent or GLP-1RA in the 90 days before meeting the inclusion criteria. Women were excluded if they were pregnant or if less than 12 months had elapsed since the first recording of pregnancy during BMI measurements. Individuals with a history of chronic pancreatitis, acute pancreatitis in the 180 days before meeting the inclusion criteria, end-stage renal disease, or renal replacement therapy (identified by primary or secondary care diagnosis codes of chronic kidney disease stage 5, dialysis, or kidney transplant) were also excluded, as were individuals with a history of malignant neoplasms in the 5 years before meeting the inclusion criteria or individuals with fatal MI or fatal stroke recorded in the 30 days before population entry.

An open cohort study design was utilised to accommodate different start and end dates (Supplementary Fig. S1 (see section on Supplementary materials given at the end of the article)). Cohort entry was the first date between 1 January 2004 and 31 December 2019 (i.e. before the COVID-19 pandemic) when all inclusion criteria were met. The start date was either the first diagnosis of CVD or the beginning of the study period if a diagnosis of CVD was recorded before the beginning of the study period. To minimise misclassification of events related to the initial diagnosis, a 30-day exclusion period was applied following cohort entry, during which any recurrent event of the same type was not considered. The end date was the earliest occurrence of death, record of transfer out of the region, or end of the study period, whichever occurred first. For the purpose of this analysis, semaglutide was assumed to be initiated for a lifetime duration.

### Outcomes and statistical analysis

Outcomes assessed in this study were life expectancy and recurrent CV event-free life-years, also assessed as life-years and CV event-free life-years gained with semaglutide versus no semaglutide treatment. The CV events investigated were MI and stroke. CV event-free life expectancy was defined as the time from entry into the study population to the occurrence of a fatal or non-fatal MI or stroke. All-cause mortality during 2004–2019 (obtained from primary care data available from 1 January 2004) and CV events during 2015–2019 (obtained from linked secondary care data available from 1 January 2015) were also analysed. The collection period for fatal and non-fatal events differed owing to the variations in the availability of primary and secondary care data in the Discover database. To align with the mean age of the SELECT trial population (61.6 years; standard deviation (SD): 8.9), outcomes are reported for individuals aged ≥45 years (14).

Outcomes were estimated for groups defined by 5-year age ranges (45–49, 50–54, 55–59, 60–64, 65–69 years, and ≥70 years). Outcomes were also estimated by sex (male or female), ethnicity (White, Asian or Asian British, Black or Black British, or other ethnicities), and multimorbidity (defined as the presence of ≥2 or ≥3 obesity-related complications (ORCs)). The ORCs considered were selected based on previous studies (16) and were as follows: asthma, atherosclerotic CVD (ASCVD), back pain, chronic kidney disease, dyslipidaemia, gastro-oesophageal reflux disease, heart failure, hypertension, obstructive sleep apnoea, osteoarthritis of the knee, polycystic ovary syndrome, prediabetes, psoriasis, and urinary incontinence. All individuals included in the study had ASCVD and were therefore considered to have at least one ORC.

**Figure 1:**
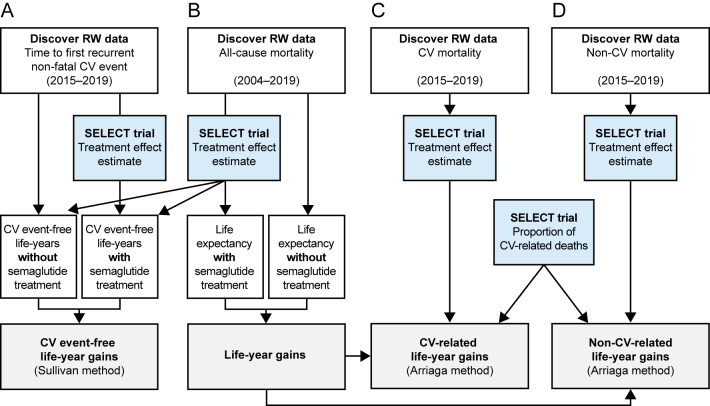
Outcomes and statistical analysis for (A) CV event-free life-year gains, (B) life-year gains, and (C) CV-related and (D) non-CV-related life-year gains. CV, cardiovascular; RW, real-world.

An overview of the outcomes and analysis methodology is shown in Fig. 1. Data from the Discover study population were first used to construct actuarial life tables for all-cause mortality; effect estimates based on SELECT trial outcomes were then applied to calculate the potential increase in life expectancy and 95% CIs with semaglutide treatment (Fig. 1B). For outcomes for which the aggregate population effect estimate was statistically significant (i.e. time to all-cause mortality, time to non-CV mortality, and time to non-fatal MI), the aggregate estimate was applied to all subgroups. For outcomes for which the aggregate population effect estimate was not statistically significant (i.e. time to CV mortality and time to non-fatal stroke), an effect estimate of 1.0 (i.e. no effect) was imputed for all subgroups (Supplementary Table 1).

The Sullivan method was used to derive CV event-free life expectancy (Fig. 1A). The Sullivan method estimates healthy life expectancy by combining age-specific mortality data with prevalence data on health states (17). To estimate the proportion of time spent without recurrent CV events for each age group, the follow-up time for each individual was converted to time before the first CV event during the study period and incorporated into the actuarial life tables. To determine the potential effect of semaglutide on life expectancy, population mortality for the treated population was determined by multiplying the calculated rates from the actuarial life tables for the untreated population by the all-cause mortality HRs. The difference between the treated and untreated life-years represents the years of life gained from treatment.

The Arriaga method was used to estimate CV- and non-CV-related mortality and life-year gains (Fig. 1C). This method interprets changes in life expectancy determined by age groups and causes of death (18). CV- and non-CV-related mortality for the untreated population was obtained using data from the Discover study population. The proportion of CV-related mortality was adjusted for in the treated population using the estimates of treatment effect for semaglutide from the SELECT trial. The effect estimate for all-cause mortality was attributed to both CV- and non-CV-related mortality to preserve the proportion of CV-related deaths. The number of years contributed to the life expectancy gap that was caused by changes in CV-related mortality was then calculated by dividing the difference in CV-specific mortality by the difference in all-cause mortality for each age group and multiplied by the overall contribution to the life expectancy gap for each age group. Total contributions of CV-related mortality to life-year gains were aggregated across age-specific contributions.

### Probabilistic model

To quantify the uncertainty in the modelled life expectancy gains associated with SELECT trial effect estimates, a probabilistic model was utilised to develop HR values from a distribution defined by the 95% CI values for all-cause mortality, non-fatal MI, and non-fatal stroke (Supplementary Table 1). In total, 10,000 independent simulations of the probabilistic model were conducted, which involved independent sampling of the effect estimates from a probability distribution. Each effect estimate was assumed to follow a log-normal distribution determined by its central estimate and 95% CI derived from the SELECT trial. In this model, the HR for non-fatal stroke was not imputed; instead, the reported effect estimates and CIs were used directly, because the sampling across iterations should account for the lack of significance.

## Results

### Study population and baseline characteristics

In total, 19,117 individuals aged ≥45 years were included (Table 1). Men comprised the majority (63%) of the study population, and the mean age was 65.4 years. Our study population was older than the SELECT trial population (14) (65.4 vs 61.6 years), with a lower proportion of men (63 vs 72%) and a higher proportion of Black (8 vs 4%) and Asian (21 vs 8%) individuals.

**Table 1:** Baseline characteristics of the study population by age group.

Age group	Overall	45–49 years	50–54 years	55–59 years	60–64 years	65–69 years	≥70 years
*n* (%)	19,117 (100.0)	2,150 (11.2)	2,367 (12.4)	2,355 (12.3)	2,570 (13.4)	2,517 (13.2)	7,158 (37.4)
Men, *n* (%)	12,124 (63.4)	1,561 (72.6)	1,754 (74.1)	1,700 (72.2)	1,843 (71.7)	1,640 (65.2)	3,626 (50.7)
BMI, kg/m^2^, mean (SD)	30.8 (4.3)	31.6 (4.7)	31.2 (4.5)	30.9 (4.3)	30.7 (4.0)	30.7 (4.1)	30.4 (4.1)
Ethnicity, *n* (%)							
White	11,809 (61.8)	1,064 (49.5)	1,259 (53.2)	1,355 (57.5)	1,609 (62.6)	1,601 (63.6)	4,921 (68.7)
Asian or Asian British	3,946 (20.6)	612 (28.5)	598 (25.3)	572 (24.3)	520 (20.2)	518 (20.6)	1,126 (15.7)
Black or Black British	1,448 (7.6)	239 (11.1)	239 (10.1)	181 (7.7)	163 (6.3)	169 (6.7)	457 (6.4)
Other ethnicities	1,161 (6.1)	150 (7.0)	183 (7.7)	158 (6.7)	185 (7.2)	148 (5.9)	337 (4.7)
Mixed	387 (2.0)	68 (3.2)	69 (2.9)	53 (2.3)	52 (2.0)	39 (1.5)	106 (1.5)
Unknown	366 (1.9)	17 (0.8)	19 (0.8)	36 (1.5)	41 (1.6)	42 (1.7)	211 (2.9)
Laboratory measurements, mean (SD)							
eGFR, mL/min/1.73 m^2^	71.8 (16.8)	80.0 (13.5)	78.3 (13.7)	76.7 (13.7)	74.7 (14.8)	72.0 (15.3)	64.5 (17.7)
HbA_1c_, %	6.0 (0.9)	6.0 (1.1)	6.0 (1.0)	6.0 (1.0)	6.0 (0.9)	6.0 (0.9)	5.9 (0.7)
DBP, mmHg	79.1 (11.0)	81.9 (11.0)	82.5 (11.2)	81.5 (10.9)	80.3 (10.4)	79.1 (10.4)	75.9 (10.6)
SBP, mmHg	135.6 (18.1)	131.0 (17.6)	133.8 (18.2)	134.4 (18.5)	135.9 (17.6)	136.7 (17.9)	137.4 (18.2)
HDL-C, mmol/L	1.3 (0.4)	1.2 (0.4)	1.2 (0.3)	1.2 (0.4)	1.3 (0.4)	1.3 (0.4)	1.4 (0.4)
LDL-C, mmol/L	2.8 (1.0)	2.9 (1.1)	3.0 (1.1)	2.9 (1.1)	2.9 (1.0)	2.8 (1.0)	2.7 (1.0)
Serum creatinine, μmol/L	87.1 (25.9)	83.1 (18.2)	83.9 (19.7)	83.6 (18.4)	84.6 (20.6)	86.2 (24.4)	91.8 (32.5)
Triglycerides, mmol/L	1.6 (1.0)	1.9 (1.3)	1.8 (1.3)	1.7 (1.0)	1.7 (1.0)	1.6 (0.9)	1.4 (0.7)
CVD,* *n* (%)							
MI only	10,519 (55.0)	1,328 (61.8)	1,510 (63.8)	1,443 (61.3)	1,478 (57.5)	1,361 (54.1)	3,399 (47.5)
Stroke only	7,842 (41.0)	774 (36.0)	795 (33.6)	807 (34.3)	976 (38.0)	1,039 (41.3)	3,451 (48.2)
PAD^†^ only	705 (3.7)	45 (2.1)	61 (2.6)	97 (4.1)	110 (4.3)	105 (4.2)	287 (4.0)
Number of ORCs,^‡^ *n* (%)							
≥2 ORCs	16,446 (86.0)	1,668 (77.6)	1,896 (80.1)	1,946 (82.6)	2,175 (84.6)	2,185 (86.8)	6,576 (91.9)
≥3 ORCs	11,042 (57.8)	932 (43.3)	1,028 (43.4)	1,174 (49.9)	1,395 (54.3)	1,491 (59.2)	5,022 (70.2)

* In total, 51 individuals had ≥2 CVD types.

^†^ Includes revascularisation procedure, claudication with ankle–brachial index <0.85, or amputation because of atherosclerotic disease.

^‡^ ORCs were asthma, ASCVD, back pain, chronic kidney disease, dyslipidaemia, gastro-oesophageal reflux disease, heart failure, hypertension, obstructive sleep apnoea, osteoarthritis of the knee, polycystic ovary syndrome, prediabetes, psoriasis, and urinary incontinence.

ASCVD, atherosclerotic cardiovascular disease; BMI, body mass index; CVD, cardiovascular disease; DBP, diastolic blood pressure; eGFR, estimated glomerular filtration rate; HbA_1c_, glycated haemoglobin; HDL-C, high-density lipoprotein cholesterol; LDL-C, low-density lipoprotein cholesterol; MI, myocardial infarction; ORC, obesity-related comorbidity; PAD, peripheral artery disease; SBP, systolic blood pressure; and SD, standard deviation.

### Remaining life-year gains in individuals treated with semaglutide

**Figure 2:**
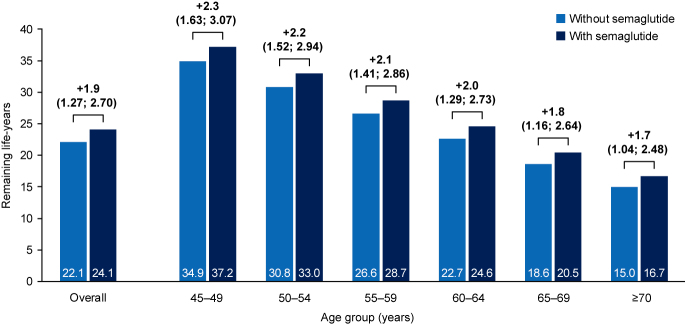
Remaining life-years with and without semaglutide by age group. The increase in life expectancy and 95% CIs (in years) observed with semaglutide for each age group is shown above the bars. CI, confidence interval.

The estimate of remaining life-years was greater for individuals treated with semaglutide than individuals not treated with semaglutide, resulting in an estimated life-year gain of 1.9 years (95% CI: 1.27–2.70) with semaglutide (Fig. 2, Supplementary Table 2). The estimated overall life expectancy gain with semaglutide treatment ranged from 2.3 years (95% CI: 1.63–3.07) in the group aged 45–49 years (*n* = 2,150) to 1.7 life-years (95% CI: 1.04–2.48) in the group aged ≥70 years (*n* = 7,158) (Fig. 2). Of this estimated gain in the groups aged 45–49 years and ≥70 years, 0.4 and 0.3 years were attributed to reductions in CV-related mortality and 1.9 and 1.1 years were attributed to reductions in non-CV-related mortality, respectively (Supplementary Fig. S2). In the probabilistic model for individuals aged 45 years, 53.4% experienced a life expectancy gain of at least 2.3 years (Supplementary Fig. S3A).

### Remaining CV event-free life-year gains in individuals treated with semaglutide

**Figure 3:**
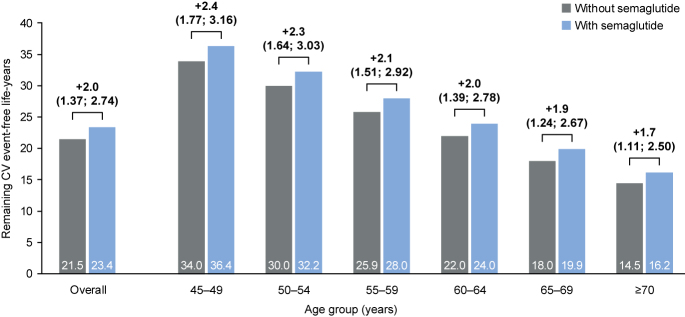
Remaining CV event-free life-years with and without semaglutide by age group. The increase in CV event-free life-years and 95% CIs (in years) observed with semaglutide for each age group is shown above the bars. CI, confidence interval; CV, cardiovascular.

The mean time to death for all individuals aged ≥45 years was 5.43 years (SD: 3.76), and the mean time to first CV event was 1.69 years (SD: 1.33). Overall, across all age groups, 2.0 CV event-free life-years (95% CI: 1.37–2.74) were estimated to be gained with semaglutide (Fig. 3, Supplementary Table 3). The estimated CV event-free life expectancy gain with semaglutide was 2.4 years (95% CI: 1.77–3.16) in the group aged 45–49 years, decreasing to 1.7 years (95% CI: 1.11–2.50) in the group aged ≥70 years (Fig. 3). In the probabilistic model for individuals aged 45 years, 55.1% experienced a CV event-free life expectancy gain of at least 2.4 years with semaglutide treatment (Supplementary Fig. S3B).

### Remaining life-year and CV event-free life-year gains in individuals treated with semaglutide stratified by sex, ethnicity, and multimorbidity

This SELECT-like study population consisted of the following ethnicities: White (61.8%), Asian or Asian British (20.6%), Black or Black British (7.6%), other ethnicities (6.1%), mixed (2.0%), and unknown (1.9%). The estimated overall life expectancy gain with semaglutide was 1.9 years for men, women, and White individuals. Similar life-year gains were observed for individuals of Asian, Black, and other ethnicities (Supplementary Table 2). The estimated CV event-free life-year gains for individuals treated with semaglutide were 2.0 years for men, women, and White individuals. Similar CV event-free life-year gains were observed for individuals of Asian, Black, and other ethnicities (Supplementary Table 3).

All individuals included in the study had ASCVD and, consequently, had at least one ORC at study entry. At baseline, 86.0 and 57.8% had ≥2 and ≥3 ORCs, respectively (Table 1). The estimated overall life expectancy gain with semaglutide treatment was 2.2 years (95% CI: 0.69–3.25) for individuals with ≥2 ORCs and 2.3 years (95% CI: 0.68–3.27) for individuals with ≥3 ORCs (Supplementary Table 2). The estimated CV event-free life-year gains for individuals treated with semaglutide were 2.3 years (95% CI: 0.80–3.28) for both groups of individuals with ≥2 ORCs and ≥3 ORCs (Supplementary Table 3).

## Discussion

In this UK real-world population of individuals aged ≥45 years with overweight or obesity and established CVD, treatment with semaglutide was estimated to increase life expectancy by 1.9 years. Semaglutide was also associated with an estimated 2.0-year increase in CV event-free life expectancy. Individuals in the younger age brackets (45–49 years, 50–54 years, and 55–59 years) experienced the greatest benefit from semaglutide treatment.

Similar to the SELECT trial, the present study had a high proportion of men (63%). It is therefore difficult to extrapolate the results to women who may have lower 10-year absolute CV risk but longer lifetimes (2, 19). Over a time frame of 5–10 years, women have a lower absolute risk of CVD and CV-related mortality than men (19, 20, 21). However, the lifetime risk of CVD and CV-related mortality is similar between women and men (22), because women tend to develop a first CV-related event later in life than men (23, 24). There are also differences in the types of events: as a first event, men are more likely to develop coronary heart disease, whereas women are more likely to develop cerebrovascular disease or heart failure (22).

In this study, life-year gains and CV event-free life-year gains were similar. This is because most of the gains in CV event-free life-years were attributed to improvements in mortality, with only a small gain due to reductions in non-fatal MI. The percentage gains in estimated CV event-free life-years were greater than the gains observed in estimated life expectancy because they were applied to a smaller baseline amount of time. Furthermore, in the SELECT trial, there was a greater risk reduction in all-cause mortality than in CV mortality (14). Questions persist as to the mechanisms for CV benefit with semaglutide compared with placebo in the SELECT trial, which may include improvement in insulin sensitivity and the potential anti-inflammatory effects of semaglutide and a decrease in conversion of prediabetes to diabetes; as such, more specific future analyses investigating similar changes in this Discover cohort are of interest.

In other modelling studies, statins have been estimated to produce life expectancy gains similar to those seen with semaglutide in this analysis (25, 26). In individuals aged ≥55 years, treatment with statins resulted in a life-year gain of 0.3 years and an increase in CVD-free life expectancy of 0.7 years compared with no statin therapy (25). In men and women aged 45 years with low-density lipoprotein cholesterol levels <149 mg/dL and C-reactive protein levels ≥1.6 mg/L, statin therapy (vs no statin therapy) was estimated to provide life-year gains of 0.8 and 0.6 years, respectively (26). These benefits have established statins as the standard of care for treatment of CVD (27, 28). The added benefits of semaglutide treatment are incremental to those obtained with statins and other underlying standard of care therapies and could be due to the effects on cardiometabolic risk factors, including obesity, fasting plasma glucose, and fasting serum insulin (29). However, the benefits observed with statins were in populations without CVD and would likely be higher in populations that were similar to the one in this study.

Our study has strengths and limitations. The use of the Discover database enabled estimations of life expectancy outcomes in a real-world, SELECT trial-like population. This analysis was based on a UK population from North West London; however, the potential estimated benefits of semaglutide treatment are likely to be applicable to other populations globally, depending on baseline risk, access to healthcare, and socioeconomic factors. The application of SELECT trial data to model outcomes in other populations is supported by the international nature of SELECT sites, the large population size, and the consistency of findings reported in SELECT across subgroups. The use of SELECT trial data has several caveats. In SELECT, 26.7% of participants who received semaglutide had discontinued treatment by the end of the trial (14). The life expectancy estimates in the present analysis accounted for these rates of discontinuation in the trial follow-up period (in accordance with the intention-to-treat principle), but not beyond this period, because these rates are unknown. Consequently, the reported life expectancy gains may overestimate the expected effect of treatment over a person’s lifetime. Furthermore, these analyses were based on the treatment effect estimates in SELECT for semaglutide, which are assumptions based on modelling of the data from the SELECT trial at a constant rate and effect over a lifetime of treatment. In this present study, individuals were not excluded by New York Heart Association class IV heart failure classification (a criteria of the SELECT trial) because these data were not directly recorded in the database; this could reduce the extent of the benefits observed by modelling, lead to biases towards extreme values, and ignore other potential effects. The study period ranged from 2004 to 2019, a time frame that may reflect changes to guidelines and standard of care, with individuals receiving less lipid-lowering management strategies at the beginning of the study period than at the end of the study period. The study period for CV events was derived from secondary care data, which were only linked from 2015. Consequently, these data reflect more recent lipid-lowering management strategies but lack data with longitudinal depth.

## Conclusion

Based on results from the SELECT trial of semaglutide in people with established CVD and no T2D, semaglutide was estimated to increase life expectancy and CV event-free life-years in a real-world population with overweight or obesity and established CVD, with even greater benefits seen in younger people (45–59 years). These results may help to inform individualised treatment decision-making.

## Supplementary materials



## Declaration of interest

TVS has received research operating funds from the Dutch Diabetes Foundation (fellowship grant number 2021.81.004) and the European Foundation for the Study of Diabetes (Boehringer Ingelheim European Research Programme on Multi-System Challenges in Diabetes). SHJH has nothing to disclose. JW is a Principal Investigator for the Netherlands in the SURPASS-CVOT trial and receives expense reimbursement for their time related to this role from Eli Lilly. SC and JDRF are employees and shareholders of Novo Nordisk A/S. SH and AP are employees of Lane Clark & Peacock LLP. NT was an employee of Lane Clark & Peacock LLP at the time of the analysis and is now an employee of the Washington State Hospital Association. Lane Clark & Peacock LLP received consulting fees from Novo Nordisk A/S to perform this analysis. SE received consulting honoraria from 89Bio, Amylyx, AstraZeneca, Avillion, Ayala, Bayer, BeiGene, BioAge, BioAtla, Boehringer Ingelheim, BridgeBio, Bristol Myers Squibb, Daiichi Sankyo, Denovo, Fore Therapeutics, GSK, Inovio, Insmed, Ipsen, Karuna, Eli Lilly, Lundbeck, Mirati, Moderna, Novartis, Novavax, Novo Nordisk, National Surgical Adjuvant Breast and Bowel Project (NSABP), Pfizer, Principia, Reata, Rebiotix, Roche, Sanofi, SOLVD Health, Sutro Biopharma, and TG Therapeutics. AML received research funding from AbbVie, AstraZeneca, CSL, Eli Lilly, Esperion, and Novartis and received consulting fees from Akebia, Alnylam, Amgen, Ardelyx, Becton, Dickinson and Company, BrainStorm, Cadrenal, Cell, Eli Lilly, Endologix, FibroGen, GSK, Intarcia, Medtronic, Neovasc, Novo Nordisk, Provention Bio, and ReCor. JP received consultancy fees from Altimmune, Amgen, Boehringer Ingelheim, Corcept, Esperion (clinical trial steering committee), Merck, NewAmsterdam Pharma, and Novo Nordisk (clinical trial steering committee, consultant); he received grant support (as principal investigator) from Boehringer Ingelheim, National Institutes of Health (NIH)/National Institute of Diabetes and Digestive and Kidney Diseases (NIDDK), and Novartis. IL received research funding (paid to institution) and/or product from Boehringer Ingelheim, Dexcom, Eli Lilly, Novo Nordisk, Pfizer, Roche; received research-related consulting fees (paid to institution) from Novo Nordisk; and served as a consultant and received consulting fees (paid to institution) from Aardvark Therapeutics, AbbVie, Alveus Therapeutics, Amgen, Antag Therapeutics, Arrowhead Pharmaceuticals, AstraZeneca, Baim Institute, Bain Capital, Bayer Healthcare Pharmaceuticals, Betagenon AB, Bioio Inc., Biomea, Boehringer Ingelheim, Boston Scientific, Carmot, Corxel, Cytoki Pharma, Eli Lilly, Genentech, Johnson & Johnson Medical Devices & Diagnostics Group – Latin America, Juvena, Keros Therapeutics, Inc, Mediflix, Metsera, Neurocrine, Novo Nordisk, Penguin Bio, Pfizer, Regeneron, Response Pharmaceutical, Roche, Sanofi, Shionogi, Skye Bioscience, Source Bio, Structure Therapeutics, SUMMIT, Tenvie, TERNS Pharma, Verdiva Bio, Viking Therapeutics, and Zealand Pharma.

## Funding

Lane Clark & Peacock LLP received funding from Novo Nordisk A/S to perform this analysis.

## Author contribution statement

AP, NT, and SH contributed to the design of the analyses. All authors contributed to data interpretation and to critical review and revision of the manuscript. All authors approved the final version of the manuscript for submission.

## Ethical approval

This study was a retrospective analysis of secondary de-identified data and was not considered to be human subject research, and therefore, NHS ethical approval was not required.

## References

[1] World Health Organization (WHO). Global health estimates: life expectancy and healthy life expectancy, 2024. (https://www.who.int/data/gho/data/themes/mortality-and-global-health-estimates/ghe-life-expectancy-and-healthy-life-expectancy). Accessed on 5 March 2025.

[2] Office for National Statistics (ONS). National life tables – life expectancy in the UK: 2021 to 2023, 2025. (https://www.ons.gov.uk/peoplepopulationandcommunity/birthsdeathsandmarriages/lifeexpectancies/bulletins/nationallifetablesunitedkingdom/2021to2023additionaldata). Accessed on 31 October 2024.

[3] Lichtenberg K. Reversing the decreasing life expectancy: a national health priority. Mo Med 2022 119 321–333. 36118821 PMC9462908

[4] Oboza P, Ogarek N, Olszanecka-Glinianowicz M, et al. The main causes of death in patients with COVID-19. Eur Rev Med Pharmacol Sci 2023 27 2165–2172. (10.26355/eurrev_202303_31589) 36930516

[5] World Health Organization (WHO). Global health estimates: life expectancy and leading causes of death and disability, 2024. (https://www.who.int/data/gho/data/themes/mortality-and-global-health-estimates). Accessed on 5 March 2025.

[6] Office for Health Improvement & Disparities (OHID). Understanding the drivers of healthy life expectancy: report, 2023. (https://www.gov.uk/government/publications/understanding-the-drivers-of-healthy-life-expectancy/understanding-the-drivers-of-healthy-life-expectancy-report#references). Accessed on 5 February 2025.

[7] Aune D, Sen A, Prasad M, et al. BMI and all cause mortality: systematic review and non-linear dose-response meta-analysis of 230 cohort studies with 3.74 million deaths among 30.3 million participants. BMJ 2016 353 i2156. (10.1136/bmj.i2156) 27146380 PMC4856854

[8] Flegal KM, Kit BK, Orpana H, et al. Association of all-cause mortality with overweight and obesity using standard body mass index categories: a systematic review and meta-analysis. JAMA 2013 309 71–82. (10.1001/jama.2012.113905) 23280227 PMC4855514

[9] Ng M, Fleming T, Robinson M, et al. Global, regional, and national prevalence of overweight and obesity in children and adults during 1980–2013: a systematic analysis for the Global Burden of Disease Study 2013. Lancet 2014 384 766–781. (10.1016/S0140-6736(14)60460-8) 24880830 PMC4624264

[10] Lopez-Jimenez F, Almahmeed W, Bays H, et al. Obesity and cardiovascular disease: mechanistic insights and management strategies. A joint position paper by the World Heart Federation and World Obesity Federation. Eur J Prev Cardiol 2022 29 2218–2237. (10.1093/eurjpc/zwac187) 36007112

[11] Bogers RP, Bemelmans WJ, Hoogenveen RT, et al. Association of overweight with increased risk of coronary heart disease partly independent of blood pressure and cholesterol levels: a meta-analysis of 21 cohort studies including more than 300 000 persons. Arch Intern Med 2007 167 1720–1728. (10.1001/archinte.167.16.1720) 17846390

[12] World Health Organization (WHO). Obesity and overweight, 2024. (https://www.who.int/news-room/fact-sheets/detail/obesity-and-overweight#:∼:text=In%202022%2C%202.5%20billion%20adults,16%25%20were%20living%20with%20obesity). Accessed on 5 March 2025.

[13] Visaria A & Setoguchi S. Body mass index and all-cause mortality in a 21st century U.S. population: A National Health Interview Survey Analysis. PLoS One 2023 18 e0287218. (10.1371/journal.pone.0287218) 37405977 PMC10321632

[14] Lincoff AM, Brown-Frandsen K, Colhoun HM, et al. Semaglutide and cardiovascular outcomes in obesity without diabetes. N Engl J Med 2023 389 2221–2232. (10.1056/NEJMoa2307563) 37952131

[15] Bottle A, Cohen C, Lucas A, et al. How an electronic health record became a real-world research resource: comparison between London’s Whole Systems Integrated Care database and the Clinical Practice Research Datalink. BMC Med Inform Decis Mak 2020 20 71. (10.1186/s12911-020-1082-7) 32312259 PMC7171852

[16] Kivimaki M, Strandberg T, Pentti J, et al. Body-mass index and risk of obesity-related complex multimorbidity: an observational multicohort study. Lancet Diabetes Endocrinol 2022 10 253–263. (10.1016/S2213-8587(22)00033-X) 35248171 PMC8938400

[17] Sullivan DF. A single index of mortality and morbidity. HSMHA Health Rep 1971 86 347–354. (10.2307/4594169) 5554262 PMC1937122

[18] Arriaga EE. Measuring and explaining the change in life expectancies. Demography 1984 21 83–96. (10.2307/2061029) 6714492

[19] Jorstad HT, Colkesen EB, Boekholdt SM, et al. Estimated 10-year cardiovascular mortality seriously underestimates overall cardiovascular risk. Heart 2016 102 63–68. (10.1136/heartjnl-2015-307668) 26261158 PMC4717404

[20] Banks E, Welsh J, Joshy G, et al. Comparison of cardiovascular disease risk factors, assessment and management in men and women, including consideration of absolute risk: a nationally representative cross-sectional study. BMJ Open 2020 10 e038761. (10.1136/bmjopen-2020-038761) PMC7757475 33371018

[21] Lv L, Rajpura J, Liu M, et al. Prevalence and clinical characteristics of patients with hsCRP testing and test-confirmed systemic inflammation among individuals with atherosclerotic cardiovascular disease with or without chronic kidney disease in the United States (PLUTUS). Am J Prev Cardiol 2025 21 100950. (10.1016/j.ajpc.2025.100950) 40060171 PMC11889733

[22] Leening MJ, Ferket BS, Steyerberg EW, et al. Sex differences in lifetime risk and first manifestation of cardiovascular disease: prospective population based cohort study. BMJ 2014 349 g5992. (10.1136/bmj.g5992) 25403476 PMC4233917

[23] Chrysohoou C, Aggeli C, Avgeropoulou C, et al. Cardiovascular disease in women: executive summary of the expert panel statement of women in cardiology of the hellenic cardiological society. Hellenic J Cardiol 2020 61 362–377. (10.1016/j.hjc.2020.09.015) 33045394 PMC7546688

[24] Maas AH & Appelman YE. Gender differences in coronary heart disease. Neth Heart J 2010 18 598–602. (10.1007/s12471-010-0841-y) 21301622 PMC3018605

[25] Ferket BS, van Kempen BJ, Heeringa J, et al. Personalized prediction of lifetime benefits with statin therapy for asymptomatic individuals: a modeling study. PLoS Med 2012 9 e1001361. (10.1371/journal.pmed.1001361) 23300388 PMC3531501

[26] Blake GJ, Ridker PM & Kuntz KM. Projected life-expectancy gains with statin therapy for individuals with elevated C-reactive protein levels. J Am Coll Cardiol 2002 40 49–55. (10.1016/s0735-1097(02)01914-9) 12103255

[27] National Institute for Health and Care Excellence (NICE). Cardiovascular disease: risk assessment and reduction, including lipid modification, 2023. (https://www.nice.org.uk/guidance/ng238/chapter/Recommendations#discussions-and-assessment-before-starting-statins). Accessed on 19 September 2025.

[28] Chou R, Cantor A, Dana T, et al. Statin use for the primary prevention of cardiovascular disease in adults: updated evidence report and systematic review for the US Preventive Services Task Force. JAMA 2022 328 754–771. (10.1001/jama.2022.12138) 35997724

[29] Kosiborod MN, Bhatta M, Davies M, et al. Semaglutide improves cardiometabolic risk factors in adults with overweight or obesity: STEP 1 and 4 exploratory analyses. Diabetes Obes Metab 2023 25 468–478. (10.1111/dom.14890) 36200477 PMC10092593

