# Supplementary material for: Projected life-year gains with semaglutide in individuals with cardiovascular disease without type 2 diabetes in the UK
Source: Endocr Connect. 2026 Mar 23;15(3):e250903. doi: 10.1530/EC-25-0903 (PMC13034523; doi:10.1530/EC-25-0903)
Supplement: Supplementary file 1 [file supplementary_materials.pdf]

Projected life-year gains with semaglutide in individuals with cardiovascular disease without type 2 diabetes in the UK

Supplementary information

Supplementary Table 1 SELECT trial HRs and 95% CI for all-cause mortality, CV mortality, non-CV mortality, non-fatal MI, and non-fatal stroke.

**Population**	**Time to event (days), HR (95% CI)**				
	All-cause mortality	CV mortality	Non-CV mortality	Non-fatal MI	Non-fatal stroke
Overall	**0.81 (0.71–0.93)**	0.85 (0.71–1.01)	**0.77 (0.62–0.95)**	**0.72 (0.61–0.85)**	0.93 (0.74–1.15)

Statistically significant effect estimates are shown in bold.

CI, confidence interval; CV, cardiovascular; HR, hazard ratio; MI, myocardial infarction.

Supplementary Table 2 Remaining life-years and estimated life-year gains with and without semaglutide treatment, overall and stratified by sex, ethnicity, and multimorbidity.

**Treatment group**	**Age group**						
	**Overall (≥45 years)**	**45–49 years**	**50–54 years**	**55–59 years**	**60–64 years**	**65–69 years**	**≥70 years**
Overall population							
Remaining life-years with semaglutide	24.1	37.2	33.0	28.7	24.6	20.5	16.7
Remaining life-years without semaglutide	22.1	34.9	30.8	26.6	22.7	18.6	15.0
Estimated life-year gain (95% CI)	+1.9 (1.27; 2.70)	+2.3 (1.63; 3.07)	+2.2 (1.52; 2.94)	+2.1 (1.41; 2.86)	+2.0 (1.29; 2.73)	+1.8 (1.16; 2.64)	+1.7 (1.04; 2.48)
Sex, men							
Remaining	24.5	36.6	32.4	28.1	24.0	19.9	16.1

**Treatment group**	**Age group**						
	**Overall (≥45 years)**	**45–49 years**	**50–54 years**	**55–59 years**	**60–64 years**	**65–69 years**	**≥70 years**
life-years with semaglutide							
Remaining life-years without semaglutide	22.6	34.3	30.3	26.1	22.1	18.1	14.5
Estimated life-year gain (95% CI)	+1.9 (0.70; 3.29)	+2.3 (0.84; 3.84)	+2.1 (0.78; 3.64)	+2.0 (0.75; 3.48)	+1.9 (0.70; 3.28)	+1.8 (0.65; 3.10)	+1.6 (0.60; 2.88)
Sex, women							
Remaining life-years with semaglutide	23.0	38.2	33.8	29.5	25.4	21.3	17.4
Remaining life-years	21.1	35.8	31.6	27.4	23.4	19.4	15.7

**Treatment group**	**Age group**						
	**Overall (≥45 years)**	**45–49 years**	**50–54 years**	**55–59 years**	**60–64 years**	**65–69 years**	**≥70 years**
without semaglutide							
Estimated life-year gain (95% CI)	+1.9 (0.68; 3.27)	+2.4 (0.83; 3.92)	+2.2 (0.79; 3.75)	+2.1 (0.75; 3.60)	+2.0 (0.71; 3.40)	+1.9 (0.66; 3.22)	+1.7 (0.61; 3.01)
Ethnicity, White							
Remaining life-years with semaglutide	23.2	36.9	32.8	28.6	24.6	20.5	16.7
Remaining life-years without semaglutide	21.3	34.5	30.6	26.5	22.7	18.7	15.0
Estimated life-year gain (95%	+1.9 (0.67; 3.30)	+2.4 (0.82; 3.93)	+2.2 (0.78; 3.74)	+2.1 (0.74; 3.58)	+1.9 (0.69; 3.38)	+1.8 (0.65; 3.20)	+1.7 (0.60; 2.98)

**Treatment group**	**Age group**						
	**Overall (≥45 years)**	**45–49 years**	**50–54 years**	**55–59 years**	**60–64 years**	**65–69 years**	**≥70 years**
CI)							
Ethnicity, Asian or Asian British							
Remaining life-years with semaglutide	27.4	39.5	35.1	30.7	26.3	22.0	18.1
Remaining life-years without semaglutide	25.5	37.3	32.9	28.7	24.4	20.2	16.4
Estimated life-year gain (95% CI)	+1.9 (0.68; 3.38)	+2.2 (0.80; 3.90)	+2.2 (0.76; 3.71)	+2.0 (0.72; 3.55)	+1.9 (0.67; 3.35)	+1.8 (0.63; 3.17)	+1.7 (0.58; 2.95)
Ethnicity, Black or Black British							
Remaining life-years with	25.5	36.7	32.6	28.2	24.3	20.7	17.1

Treatment group	Age group						
	**Overall (≥45 years)**	**45–49 years**	**50–54 years**	**55–59 years**	**60–64 years**	**65–69 years**	**≥70 years**
semaglutide							
Remaining life-years without semaglutide	23.6	34.4	30.5	26.1	22.3	18.9	15.5
Estimated life-year gain (95% CI)	+1.9 (0.72; 3.41)	+2.3 (0.85; 3.93)	+2.1 (0.80; 3.74)	+2.1 (0.76; 3.58)	+2.0 (0.71; 3.38)	+1.8 (0.67; 3.20)	+1.6 (0.62; 2.98)
Ethnicity, Other							
Remaining life-years with semaglutide	28.1	41.1	36.4	31.5	26.9	22.6	18.4
Remaining life-years without semaglutide	26.1	39.0	34.3	29.4	24.9	20.7	16.6

**Treatment group**	**Age group**						
	**Overall (≥45 years)**	**45–49 years**	**50–54 years**	**55–59 years**	**60–64 years**	**65–69 years**	**≥70 years**
Estimated life-year gain (95% CI)	+2.0 (0.70; 3.40)	+2.1 (0.83; 3.92)	+2.1 (0.78; 3.73)	+2.1 (0.75; 3.57)	+2.0 (0.70; 3.37)	+1.9 (0.66; 3.19)	+1.8 (0.60; 2.97)
Individuals with ≥2 ORCs							
Remaining life-years with semaglutide	23.9	37.1	32.9	28.6	24.6	20.4	16.7
Remaining life-years without semaglutide	21.7	34.8	30.7	26.5	22.6	18.6	15.0
Estimated life-year gain (95% CI)	+2.2 (0.69; 3.25)	+2.3 (0.84; 3.88)	+2.2 (0.79; 3.68)	+2.1 (0.75; 3.52)	+2.0 (0.70; 3.31)	+1.8 (0.66; 3.14)	+1.7 (0.61; 2.91)
Individuals with ≥3 ORCs							

**Treatment group**	**Age group**						
	**Overall (≥45 years)**	**45–49 years**	**50–54 years**	**55–59 years**	**60–64 years**	**65–69 years**	**≥70 years**
Remaining life-years with semaglutide	22.9	36.6	32.6	28.5	24.5	20.4	16.5
Remaining life-years without semaglutide	20.6	34.3	30.4	26.4	22.6	18.5	14.8
Estimated life-year gain (95% CI)	+2.3 (0.68; 3.27)	+2.3 (0.86; 4.01)	+2.2 (0.80; 3.77)	+2.1 (0.75; 3.56)	+1.9 (0.70; 3.35)	+1.9 (0.66; 3.17)	+1.7 (0.61; 2.96)

CI, confidence interval; ORC, obesity-related complication.

Supplementary Table 3 Remaining CV event-free life-years and estimated CV event-free life-year gains with and without semaglutide treatment, overall and stratified by sex, ethnicity, and multimorbidity.

**Treatment group**	**Age group**						
	**Overall (≥45 years)**	**45–49 years**	**50–54 years**	**55–59 years**	**60–64 years**	**65–69 years**	**≥70 years**
Overall population							
Remaining CV event-free life-years with semaglutide	23.4	36.4	32.2	28.0	24.0	19.9	16.2
Remaining CV event-free life-years without semaglutide	21.5	34.0	30.0	25.9	22.0	18.0	14.5
Estimated CV event-free life-year gain (95% CI)	+2.0 (1.37; 2.74)	+2.4 (1.77; 3.16)	+2.3 (1.64; 3.03)	+2.1 (1.51; 2.92)	+2.0 (1.39; 2.78)	+1.9 (1.24; 2.67)	+1.7 (1.11; 2.50)
Sex, men							
Remaining CV event-free life-years with semaglutide	23.9	35.8	31.7	27.4	23.4	19.3	15.6

Remaining CV event-free life-years without semaglutide	21.9	33.4	29.5	25.3	21.4	17.5	13.9
Estimated CV event-free life-year gain (95% CI)	+2.0 (0.81; 3.31)	+2.4 (0.98; 3.91)	+2.2 (0.92; 3.69)	+2.1 (0.87; 3.52)	+2.0 (0.81; 3.30)	+1.8 (0.75; 3.12)	+1.7 (0.68; 2.88)
Sex, women							
Remaining CV event-free life-years with semaglutide	22.4	37.3	33.0	28.8	24.7	20.7	16.9
Remaining CV event-free life-years without semaglutide	20.5	34.9	30.7	26.6	22.7	18.8	15.1
Estimated CV event-free life-year gain (95% CI)	+2.0 (0.79; 3.29)	+2.4 (1.00; 3.99)	+2.3 (0.94; 3.81)	+2.2 (0.88; 3.63)	+2.0 (0.83; 3.42)	+1.9 (0.77; 3.23)	+1.8 (0.70; 3.01)
Ethnicity, White							
Remaining CV event-free life-years with semaglutide	22.7	36.1	32.1	28.0	24.1	20.0	16.3
Remaining CV event-free life-years	20.7	33.7	29.8	25.8	22.0	18.1	14.5

without semaglutide							
Estimated CV event-free life-year gain (95% CI)	+2.0 (0.79; 3.31)	+2.4 (0.98; 3.99)	+2.3 (0.92; 3.79)	+2.2 (0.87; 3.61)	+2.1 (0.81; 3.39)	+1.9 (0.75; 3.21)	+1.8 (0.69; 2.97)
Ethnicity, Asian or Asian British							
Remaining CV event-free life-years with semaglutide	26.6	38.5	34.2	29.9	25.5	21.3	17.4
Remaining CV event-free life-years without semaglutide	24.6	36.3	32.0	27.8	23.5	19.4	15.6
Estimated CV event-free life-year gain (95% CI)	+2.0 (0.81; 3.40)	+2.2 (0.97; 3.95)	+2.2 (0.91; 3.75)	+2.1 (0.85; 3.57)	+2.0 (0.79; 3.36)	+1.9 (0.74; 3.17)	+1.8 (0.67; 2.94)
Ethnicity, Black or Black British							
Remaining CV event-free life-years with semaglutide	24.5	35.5	31.5	27.1	23.3	19.7	16.3
Remaining CV event-free life-years without semaglutide	22.5	33.0	29.2	24.9	21.2	17.8	14.6

Estimated CV event-free life-year gain (95% CI)	+2.1 (0.84; 3.44)	+2.5 (1.01; 4.00)	+2.3 (0.95; 3.79)	+2.2 (0.89; 3.62)	+2.1 (0.83; 3.40)	+1.9 (0.77; 3.21)	+1.7 (0.70; 2.98)
Ethnicity, Other							
Remaining CV event-free life-years with semaglutide	27.3	40.1	35.4	30.6	26.1	21.9	17.8
Remaining CV event-free life-years without semaglutide	25.2	37.8	33.2	28.4	24.0	19.9	16.0
Estimated CV event-free life-year gain (95% CI)	+2.1 (0.83; 3.44)	+2.3 (1.00; 4.00)	+2.2 (0.94; 3.80)	+2.2 (0.88; 3.62)	+2.1 (0.82; 3.40)	+2.0 (0.76; 3.21)	+1.8 (0.70; 2.97)
Individuals with ≥2 ORCs							
Remaining CV event-free life-years with semaglutide	23.3	36.2	32.1	27.9	23.9	19.8	16.1
Remaining CV event-free life-years without semaglutide	21.0	33.8	29.9	25.8	21.9	18.0	14.4
Estimated CV event-free life-year gain	+2.3 (0.80;	+2.4 (1.01;	+2.2 (0.94;	+2.1 (0.88;	+2.0 (0.82;	+1.8 (0.77;	+1.7 (0.70;

(95% CI)	3.28)	3.96)	3.74)	3.56)	3.34)	3.16)	2.92)
Individuals with ≥3 ORCs							
Remaining CV event-free life-years with semaglutide	22.2	35.7	31.8	27.8	23.8	19.8	16.0
Remaining CV event-free life-years without semaglutide	19.9	33.3	29.6	25.6	21.8	17.9	14.2
Estimated CV event-free life-year gain (95% CI)	+2.3 (0.80; 3.28)	+2.4 (1.03; 4.07)	+2.2 (0.96; 3.81)	+2.2 (0.90; 3.60)	+2.0 (0.83; 3.37)	+1.9 (0.77; 3.18)	+1.8 (0.70; 2.96)

CI, confidence interval; CV, cardiovascular; ORC, obesity-related complication.

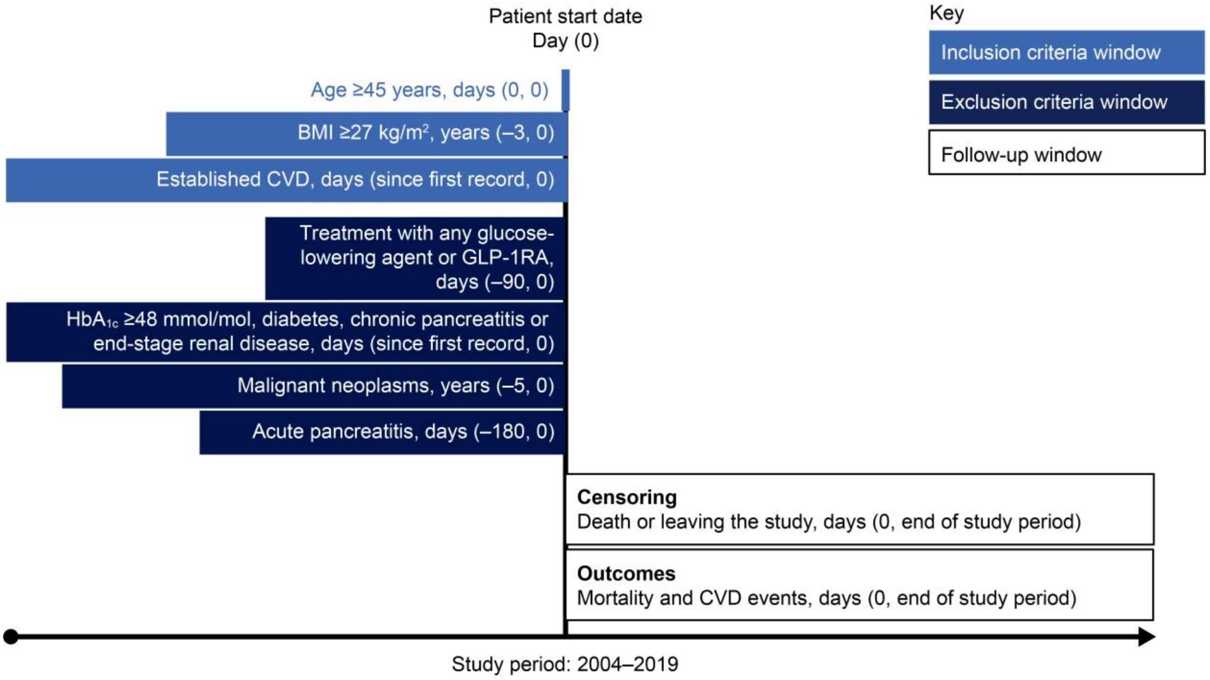

Supplementary Figure S1 Study design. BMI, body mass index; CVD, cardiovascular disease; GLP-1RA, glucagon-like peptide-1 receptor agonist; HbA_{1c}, glycated haemoglobin.

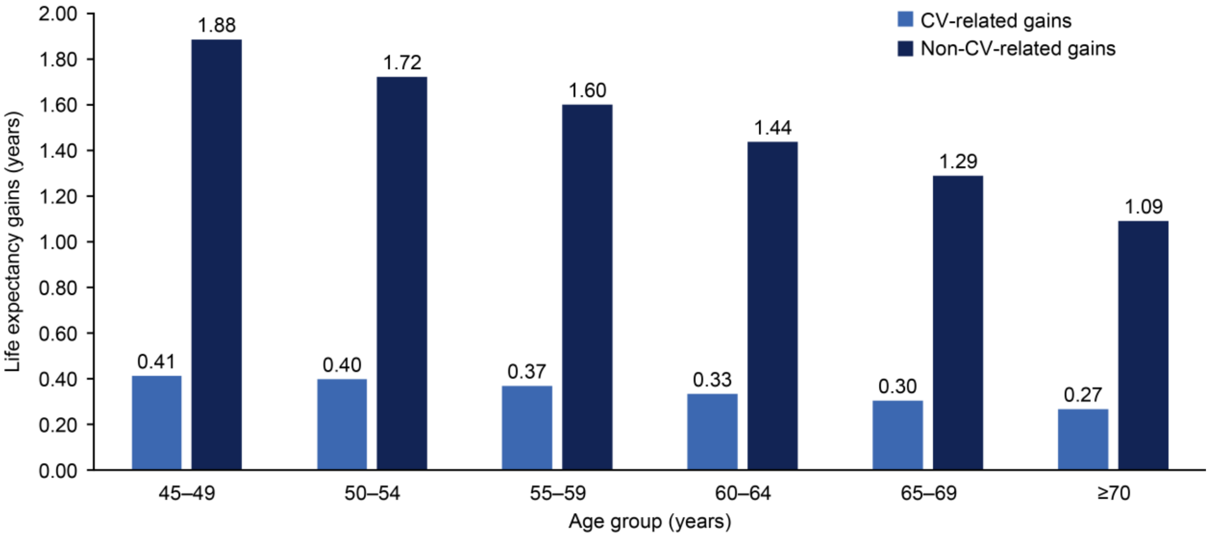

Supplementary Figure S2 Life expectancy gains associated with CV-related compared with non-CV-related gains. CV, cardiovascular.

A

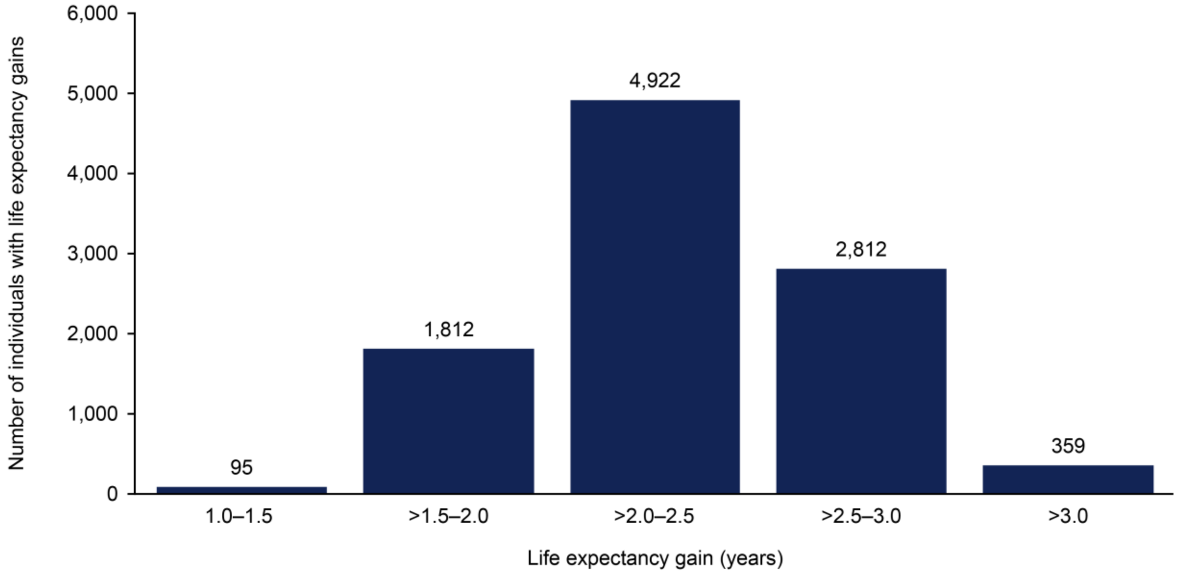

B

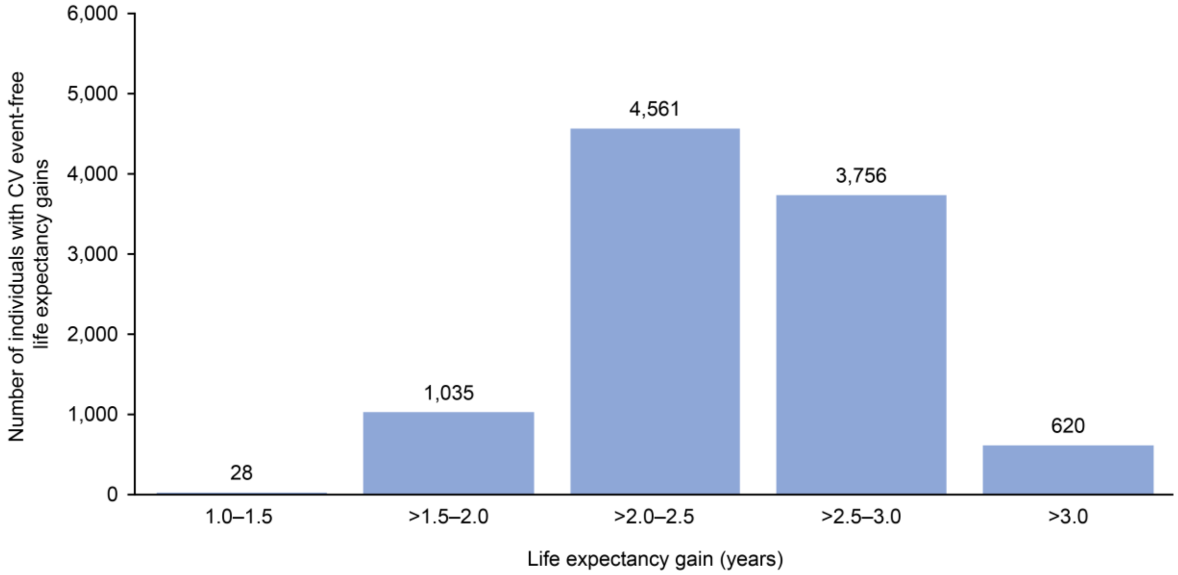

Supplementary Figure S3 Probabilistic (A) life expectancy gains and (B) CV event-free life expectancy gains with semaglutide treatment in individuals at 45 years old.

CV, cardiovascular.
